# Factors Influencing the Adoption of Contact Tracing Applications: Protocol for a Systematic Review

**DOI:** 10.2196/28961

**Published:** 2021-06-01

**Authors:** Kiemute Oyibo, Kirti Sundar Sahu, Arlene Oetomo, Plinio Pelegrini Morita

**Affiliations:** 1 School of Public Health Sciences Faculty of Health University of Waterloo Waterloo, ON Canada; 2 Institute of Health Policy, Management, and Evaluation University of Toronto Toronto, ON Canada; 3 Department of Systems Design Engineering University of Waterloo Waterloo, ON Canada; 4 eHealth Innovation, Techna Institute University Health Network Toronto, ON Canada

**Keywords:** contact tracing app, technology acceptance, user perception, adoption, COVID-19, review, perception, barrier, challenge, effective, usability

## Abstract

**Background:**

Following the onset of the COVID-19 pandemic, digital contact tracing apps have become prevalent worldwide in a coordinated effort to curb the spread of COVID-19. However, their uptake has been low and slow due to privacy concerns, the lack of trust and motivational affordances, and their minimalist design.

**Objective:**

The objective of this article is to present a protocol for a systematic review of the main factors, including facilitators and barriers, that influence the adoption of contact tracing apps.

**Methods:**

We searched seven databases, namely, Scopus, CINAHL, PubMed (MEDLINE), IEEE Xplore Digital Library, Association for Computing Machinery (ACM) Digital Library, Web of Science, and Google Scholar, for relevant publications between October 30, 2020, and January 31, 2021. Three authors were involved in removing duplicates, screening, and selection of relevant articles according to the PRISMA-P (Preferred Reporting Items for Systematic Review and Meta-analysis Protocols) guidelines.

**Results:**

Altogether, we retrieved 777 articles from the seven databases. As of May 14, 2021, we have completed the screening process and arrived at 13 eligible articles to be included in the systematic review. We hope to elicit, summarize, and report the main findings in the systematic review article by the end of August 2021. We expect to uncover facilitators and barriers related to app utility, data security, ease of use, and persuasive design that are deemed important to adoption of contact tracing apps.

**Conclusions:**

The findings of the systematic review will help researchers to uncover the gaps in the adoption of contact tracing apps, and decision makers and designers to focus on the principal adoption factors necessary to create better and more effective contact tracing apps.

**International Registered Report Identifier (IRRID):**

DERR1-10.2196/28961

## Introduction

### Rationale

Digital contact tracing apps have become prevalent worldwide due to the COVID-19 pandemic, which began in the first quarter of 2020. Specifically, these apps have been developed to fast-track the identification and self-isolation of individuals exposed to SARS-CoV-2, the coronavirus known to cause COVID-19, by being in close contact with infected persons. Several factors hinder the uptake of contact tracing apps, as is suggested in the gray and academic literature; these include privacy concerns, lack of trust, and poor persuasive design [1]. Moreover, there is limited understanding of the overall factors that influence user acceptance and adoption of such apps. According to Thorneloe et al [2], “There is a dearth of evidence [especially based on systematic reviews] regarding the barriers and facilitators to uptake and engagement with COVID-19 digital contact tracing applications.” There are several systematic reviews from the early stages of the pandemic, but they are unrelated to technology acceptance. Specifically, some systematic reviews, such as those by Braithwaite et al [3], Davalbhakta et al [4], and Juneau et al [5], did not address motivators and barriers to adoption of contact tracing apps. Hence, a systematic review of the existing literature is necessary to understand users’ perceptions, including the factors that facilitate their adoption of contact tracing apps, barriers and challenges to contact tracing app adoption, measures undertaken to tackle existing challenges, and the moderating effect of demographic or human factors. The findings of such a systematic review will uncover available opportunities to improve the design, adoption, and effectiveness of contact tracing apps in future iterations. Given the concerns and intense debate about COVID-19 contact tracing apps among governments, media, the research community, and the wider population across the globe, we surmise that privacy concern, trust, and data security will play a significant role in the adoption of contact tracing apps [6-8]. In addition, we envisage the impact of persuasive design (eg, reward and self-monitoring) on contact tracing app adoption [9]. A number of digital health researchers [10,11] have advocated the need to redesign contact tracing apps as persuasive technologies to improve their motivational appeal and overall user experience. Hence, we surmise that the incorporation of persuasive features into contact tracing apps has the potential of improving their uptake among potential users. Thus, a systematic review will reveal how relevant these empirical constructs (related to trust, privacy, security, and persuasive design) are to the adoption of contact tracing apps within and across different demographics globally.

### Objectives

This protocol, which follows the PRISMA-P (Preferred Reporting Items for Systematic Review and Meta-analysis Protocols) guidelines [12], serves as a basis for our ongoing systematic review, which sets out to identify empirical studies conducted on the acceptance and adoption of contact tracing apps since the onset of the COVID-19 pandemic. The systematic review aims to synthesize and present the empirical studies’ findings to contact tracing app stakeholders, including researchers, designers, and policymakers to improve the design of future contact tracing app iterations. Particularly, the systematic review aims to answer the following research questions (RQs):

- RQ1: What are the key facilitators and barriers that are associated with the adoption of contact tracing apps?
- RQ2: What motivational strategies are being implemented to increase the adoption of contact tracing apps?
- RQ3: What are the adoption rates of contact tracing apps among their target audiences?

## Methods

### Eligibility Criteria

For inclusion in the systematic review, the work must be a user study (quantitative, qualitative, or both) evaluating the facilitators and barriers associated with contact tracing app adoption and particular outcomes such as the intention to download, install, or use the app. The evaluation of contact tracing app must be conducted among participants from any countries around the world aged 18 years and above. These user studies must have been conducted within the period of the COVID-19 pandemic, meaning studies conducted earlier than 2020 were not considered. Moreover, the studies must be about the acceptance and adoption of contact tracing apps; that is, they can be either an evaluation of a hypothetical (described) contact tracing app or a prototype. Above all, all articles must be in English and peer reviewed. Articles that do not meet these criteria, for example, those that do not discuss facilitators and/or barriers to contact tracing app adoption, do not evaluate the adoption of contact tracing apps among participants, are not about COVID-19 contact tracing apps, are part of the gray literature, or are not written in English, will be excluded from the systematic review.

### Information Sources

Seven databases, namely Scopus, CINAHL, PubMed (MEDLINE), IEEE Xplore Digital Library, Association for Computing Machinery (ACM) Digital Library, Web of Science, and Google Scholar, were searched. The first six databases were searched between October 30, 2020, and November 20, 2020, by using the following keywords: (“contact tracing” OR “contact-tracing” OR “exposure notification” OR “exposure-notification” OR “contact notification” OR “contact-notification” OR GAEN) AND (app OR apps OR application* OR technology* OR system OR systems) AND (percept* OR adopt* OR accept* OR uptake OR use OR usage) AND (covid* OR coronavirus OR SARS-CoV-2). The criterion used for the search was “ALL” (title, abstract, keyword, and full text). Furthermore, we searched Google Scholar for articles published between November 21, 2020, and January 31, 2021, to include any additional articles that we might have missed during our systematic search conducted before November 21, 2020.

### Search Strategy

**Figure 1:**
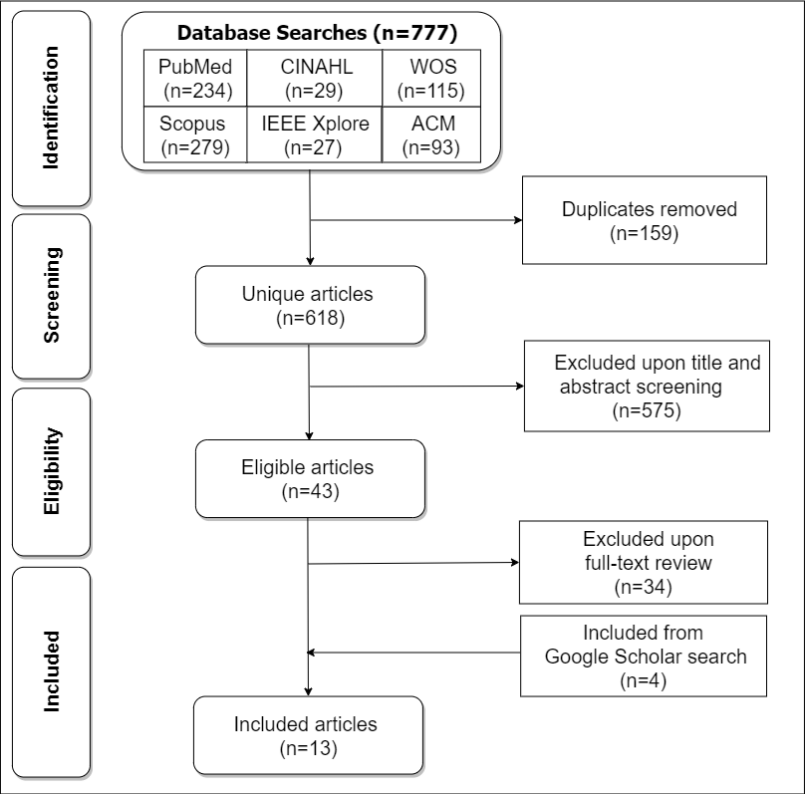
PRISMA (Preferred Reporting Items for Systematic Review and Meta-analysis) flowchart for the screening and inclusion of articles in the systematic review. ACM: Association for Computing Machinery; WOS: Web of Science.

Figure 1 shows our search strategy for identifying, screening, and including relevant articles in our systematic review. The search strategy is based on the PRISMA (Preferred Reporting Items for Systematic Review and Meta-analysis) flowchart [13]. Two approaches were used in searching for articles to be included in the systematic review. The first approach was formal (systematic) and the second was informal (nonsystematic). In the formal approach, we searched six databases (PubMed, CINAHL, Web of Science, Scopus, IEEE Xplore, and ACM Digital Library) systematically, and retrieved 777 articles in total between October 30, 2020, and November 20, 2020.

In the screening phase, we removed 159 duplicates to arrive at 618 articles. In the eligibility phase, we screened out 575 articles to arrive at 43 articles. In the inclusion phase, we excluded 34 articles upon full-text review to arrive at 9 articles.

Second, in the informal approach, between November 21, 2020, and January 31, 2021, we searched Google Scholar from time to time to uncover more articles that might not have come up in the first phase of systematic search of the six databases between October 30, 2020, and November 20, 2020. By the end of January 31, 2021, altogether, we found 4 articles from the Google Scholar search, which we added to the initial 9 articles from the systematic search to arrive at 13 articles in total for the final systematic review.

### Data Management

All articles retrieved from the non–Google Scholar databases were imported into Mendeley reference management system using Mendeley Web Importer, which was added to the Google Chrome browser. Upon merging all of the articles from the non–Google Scholar databases on Mendeley, we exported them to a Microsoft Excel spreadsheet to remove duplicates, screen the unique articles, and select the final eligible articles. Finally, we added the articles retrieved from the Google Scholar search to the final eligible articles we arrived at, according to the PRISMA screening and selection flowchart (Figure 1). In the next stage of the review process, the relevant data characteristics (eg, author names, year of publication, type of study, facilitators, and barriers) will be extracted from the 13 included articles by the three authors after a full-text reading and review, and the results will be tabulated in a Microsoft Excel spreadsheet for final systematic analysis and synthesis.

### Selection Process

Three authors initiated the screening, selection, and review of the articles retrieved from the non–Google Scholar databases and then proceeded to removing duplicates. Each author reviewed and screened an approximately equal number of articles based on titles and/or abstracts, excluding those that did not meet the eligibility criteria (see Figure 1). Articles that remained to be considered were labeled “maybe.” Next, all three authors collaborated to determine eligibility of each of the articles labeled “maybe” for possible inclusion in the full-text review. Thereafter, each of the three authors carried out a full-text review on one-third of the eligible articles to arrive at 9 articles after excluding articles that did not meet the inclusion criteria. Finally, with the 4 articles retrieved from the Google Scholar search by the first author added, the three authors reviewed the final set of included articles collectively based on the inclusion criteria to confirm and validate their inclusion in the final systematic analysis and synthesis.

### Data Collection Process

The PRISMA flowchart [13] was used to arrive at the 13 articles to be included in the systematic review. Going forward, each of the three authors will go through approximately one-third of the 13 articles to extract the key themes of interest and their corresponding values after reading the full text. When writing the systematic review article, the first author will confirm all of the extracted values by referring to the original articles, if need be.

### Data Items

The themes of interest and the respective values that will be extracted from each article are shown in Table 1. These include author identification, study date, type of application, target audience, among other themes. For example, the author identification entails values such as the name of the authors and their citations. Moreover, the target audience comprises values such as the country of the target (studied) population, the sample size, and the average age of the population.

**Table 1:** Systematic analysis coding scheme.

Number	Criterion	Description
1	Identification	Name of authors
2	Study date	Month and year
3	Type of application	Description based, prototype
4	Target audience	Country, sample size, age
5	Type of study	Quantitative, qualitative, mixed
6	Outcome variable	Intention to download app, intention to install app, intention to use app, etc
7	Facilitators	Perceived usefulness, perceived trust, self-monitoring, etc
8	Barriers	Privacy concern, perceived technology risk, etc
9	Moderating variables	Age, gender, culture, etc
10	Findings or takeaways	Summary of the main findings and takeaways
11	Recommendations	Proposed guidelines for effective design of contact tracing apps
12	Opportunities for future studies	Suggested areas for future research based on the limitations of the study

### Outcomes and Prioritization

As shown in Table 1, in the systematic review, we will search for behavioral outcomes such as the intention to download, install, or use the app. That is, in answering the third research question, “What are the adoption rates of contact tracing apps among their target audience?” for each of the empirical studies, we will be looking at, as an example, the overall rate regarding the intention to download, install, or use the contact tracing app. For instance, the percentage of study participants (especially nonadopters of contact tracing apps) who are willing to download the app under evaluation may be an indication of how the app may fair in real-life contexts, especially if the facilitators of contact tracing app adoption are prioritized. In addition, we will be looking at the levels of perception of key facilitators (eg, perceived usefulness [14]) and barriers (eg, privacy concern [15]) associated with contact tracing app adoption. For example, in a given study, “Did the participants perceive the contact tracing app described or prototyped as useful, trustworthy, etc?” We are interested in the overall scores of these constructs because, in the context of technology acceptance model, they have been found to be significant predictors or determinants of information system adoption [16-18]. Moreover, with regard to the first research question, “What are the key facilitators and barriers that are associated with the adoption of contact tracing apps?” we will be looking at studies that analyzed path models [19], structural equation models [20], or regression models. Particularly, we will be looking at the strongest predictors and the amount of variance in the outcome variables (eg, intention to use the app) explained by the predictors. The variance metric (ie, coefficient of determination) will help us to understand the extent to which the factors that have been identified in the gray literature and empirically studied are able to explain the various outcome variables associated with contact tracing app adoption.

### Data Syntheses

Using the three nonquantitative approaches, which include tabulation, graphical, and narrative approaches [21], we will synthesize the results of the empirical studies among those included in the systematic review. Moreover, we will carry out quantitative computation of metrics such as the percentage of studies that found that a given construct (eg, perceived usefulness) is a determinant of contact tracing app adoption. Finally, we will tabulate the adoption rate (ie, the percentage of participants in each study that are willing to adopt (eg, download, install, or use) a given contact tracing app under investigation.

## Results

In this section, we present the tables and diagrams of the expected results from the systematic review. Table 2 addresses the first and second research questions. It shows the tabulation of the facilitators (which may comprise persuasive or motivational strategies) and barriers associated with the adoption of contact tracing apps in each included article. The facilitators and barriers will be organized into logical categories such as app utility, data security, facilitating conditions, app design, and ethical concerns. A positive sign (+) indicates a facilitator driving contact tracing app adoption and a negative sign (–) indicates a barrier hindering contact tracing app adoption (Table 2). A typical example of a facilitator and barrier in the app utility category is “perceived usefulness” and “doubt about effectiveness,” respectively. Moreover, in the data security category, a typical example of a facilitator and barrier is “perceived trust” and “privacy concern,” respectively. For example, Velicia-Martin et al [14] and Walrave et al [15] found that perceived usefulness (a facilitator) and privacy concern (a barrier), respectively, are associated with contact tracing app adoption. Finally, “%Total” indicates the percentage of the total reviewed articles that found a given factor (facilitator or barrier) to be (1) a significant determinant of contact tracing app adoption in quantitative studies involving correlational, regression, and path analyses and/or (2) a noteworthy theme in qualitative studies based on the thematic analysis of participants’ comments.

**Table 2:** Systematic tabulation of factors and barriers associated with contact tracing app adoption. A positive sign (+) indicates a facilitator and a negative sign (–) indicates a barrier.

Category and factor		Author 1	Author 2	…	Author N	% Total
**Category 1**						
	Factor 1	+	N/A^a^	…	+	N1
	Factor 2	–	–	…	–	N2
	…	N/A	+	…	+	N3
	Factor N	+	+	…	N/A	N4
**Category 2**						
	Factor 1	+	N/A	…	+	N5
	Factor 2	–	–	…	–	N6
	…	N/A	+	…	+	N7
	Factor N	+	+	…	N/A	N8
**Category N**						
	Factor 1	+	N/A	…	+	N9
	Factor 2	–	–	…	–	N10
	…	N/A	+	…	+	N11
	Factor N	+	+	…	N/A	N12

^a^N/A: not applicable.

**Figure 2:**
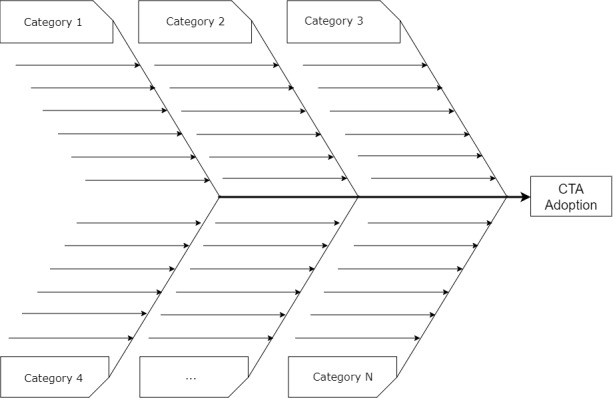
Preliminary fishbone diagram showing the factors that influence the adoption of contact tracing apps. Arrows represent facilitators and barriers. CTA: contact tracing app.

Moreover, we aim to synthesize, visually and concisely, all of the findings (facilitators and barriers associated with contact tracing app adoption) as shown in the fishbone diagram in Figure 2. Similar to Table 2, the main factors that drive and hamper contact tracing app adoption are organized into logical categories such as app utility, data security, facilitating conditions, and ethical concerns. The fishbone diagram would help readers identify the factors of contact tracing app visually in one fell swoop without the interference of overwhelming details shown in Table 2. Finally, the fishbone diagram would serve as the main takeaway of the systematic review, which, given its portability, can easily be distributed among COVID-19 contact tracing app researchers and stakeholders.

Finally, Table 3 addresses the third research question aimed at uncovering the adoption rate of contact tracing apps in each of the articles included in the systematic review. The outcome variables may include target constructs such as intention to download, install, or use the app. The adoption rate metric (especially among contact tracing app nonadopters) will provide us with insights into the percentage of participants in a given national population that may be willing to adopt contact tracing apps to curb the spread of the virus compared with the percentage of participants in another national population.

**Table 3:** Percentage of participants in each study that were willing to adopt contact tracing apps.

Variable	Author 1	Author 2	…	Author N
Country	Country 1	Country 2	…	Country N
Outcome variable (OC)	OC1	OC2	….	OCN
Adoption rate (%)	N1	N1	…	NN

## Discussion

### Directions

We have presented the protocol for a systematic review of the main factors that influence the adoption of contact tracing apps. This review is necessary in the light of the low adoption rate reported for the contact tracing apps currently available on the market worldwide. Based on an informal (nonsystematic) combing of the literature, we hope to uncover facilitators and barriers to contact tracing app adoption, which revolve around app utility, ease of use, data security, and motivational or persuasive features. Our systematic review aims to make several contributions to the existing literature. For example, the fishbone-diagram framework (Figure 2), created to visually display the results, will allow the target audience to quickly and holistically identify the primary factors that influence contact tracing app adoption. It will serve as an overarching framework for presenting the key facilitators and barriers associated with the adoption of contact tracing apps to stakeholders, including public health authorities, researchers, designers, governments, and policymakers. Overall, the findings of the systematic review will help (1) researchers to uncover the gaps in contact tracing app adoption and address them in future research efforts, and (2) decision-makers and designers to focus on the principal adoption factors necessary to create better and more effective contact tracing apps that have the potential of increasing adoption among their target audiences. We hope to complete the tabulation of our results and writing the systematic review article, which will report the main findings, takeaways, and the lessons learned, by the end of August 2021.

### Limitations

Our systematic review is subject to a number of limitations such as those arising from the risk of bias of the individual studies. Hence, in the review, individual studies will be analyzed for the risk of bias at the study level and overall. For example, for each of the studies, we hope to uncover limitations regarding small sample size and convenience sampling, which may affect the generalization of the study’s findings to the larger target audience studied. The second risk of bias is the type of quantitative analysis conducted in the studies; for example, considering the relationships between the study variables, we will determine whether it is a regression analysis, path analysis, structural equation modeling, or correlational analysis. A correlational analysis is most likely to uncover a significant relationship between two variables given that the analysis is bivariate and not multivariate. According to Kaspar [22], many of the significant relationships between variables disappear in regression and path models that consider all independent variables simultaneously. Therefore, in the systematic analysis, we will identify studies with findings based on this type of analysis that can cause biased results. The third potential source of bias in the individual studies is the adoption rate metric, which may not be representative of the actual percentage among the population under study. For example, if a study found that 50% of the participants were willing to download a given contact tracing app if it were deployed in real life, this might not reflect the actual percentage of willing adopters in the wider population. The reason is that the participants of the study in question might be more technologically literate, educated, and well-informed about the utility of contact tracing apps than the wider population. Hence, they are more likely to adopt the contact tracing app under study compared with the average person in the general population who is less informed and, thus, more likely to be susceptible to misinformation about COVID-19.

## Abbreviations

ACM: Association for Computing Machinery
COVID: coronavirus disease
GAEN: Google/Apple Exposure Notification
PRISMA: Preferred Reporting Items for Systematic Review and Meta-analysis
PRISMA-P: Preferred Reporting Items for Systematic Review and Meta-analysis Protocols
RQ: research question
WOS: Web of Science
